# Targeting TWIST1 through loss of function inhibits tumorigenicity of human glioblastoma

**DOI:** 10.1002/1878-0261.12320

**Published:** 2018-05-29

**Authors:** Andrei M. Mikheev, Svetlana A. Mikheeva, Liza J. Severs, Cory C. Funk, Lei Huang, José L. McFaline‐Figueroa, Jeanette Schwensen, Cole Trapnell, Nathan D. Price, Stephen Wong, Robert C. Rostomily

**Affiliations:** ^1^ Department of Neurosurgery Houston Methodist Hospital and Research Institute Houston TX USA; ^2^ Department of Neurosurgery and Institute for Stem Cell and Regenerative Medicine University of Washington Seattle WA USA; ^3^ Department of Physiology and Biophysics University of Washington Seattle WA USA; ^4^ Institute for Systems Biology Seattle WA USA; ^5^ Department of Systems Medicine& Bioengineering Houston Methodist Hospital and Research Institute Weil Cornell Medical College Houston TX USA; ^6^ Department of Genome Sciences University of Washington Seattle WA USA

**Keywords:** AKT, glioblastoma, glioma stem cells, periostin, tumorigenicity, TWIST1

## Abstract

TWIST1 (TW) is a bHLH transcription factor (TF) and master regulator of the epithelial‐to‐mesenchymal transition (EMT). *In vitro*, TW promotes mesenchymal change, invasion, and self‐renewal in glioblastoma (GBM) cells. However, the potential therapeutic relevance of TW has not been established through loss‐of‐function studies in human GBM cell xenograft models. The effects of TW loss of function (gene editing and knockdown) on inhibition of tumorigenicity of U87MG and GBM4 glioma stem cells were tested in orthotopic xenograft models and conditional knockdown in established flank xenograft tumors. RNAseq and the analysis of tumors investigated putative TW‐associated mechanisms. Multiple bioinformatic tools revealed significant alteration of ECM, membrane receptors, signaling transduction kinases, and cytoskeleton dynamics leading to identification of PI3K/AKT signaling. We experimentally show alteration of AKT activity and periostin (POSTN) expression *in vivo* and/or *in vitro*. For the first time, we show that effect of TW knockout inhibits AKT activity in U87MG cells *in vivo* independent of PTEN mutation. The clinical relevance of TW and candidate mechanisms was established by analysis of the TCGA and ENCODE databases. TW expression was associated with decreased patient survival and LASSO regression analysis identified POSTN as one of top targets of TW in human GBM. While we previously demonstrated the role of TW in promoting EMT and invasion of glioma cells, these studies provide direct experimental evidence supporting protumorigenic role of TW independent of invasion *in vivo* and the therapeutic relevance of targeting TW in human GBM. Further, the role of TW driving POSTN expression and AKT signaling suggests actionable targets, which could be leveraged to mitigate the oncogenic effects of TW in GBM.

**Abbreviations**
AKT: total AKT serine/threonine kinase
GBM: glioblastoma multiforme
PI3K: phosphoinositide‐3‐kinase
POSTN: periostin
PTEN: phosphatase and tensin homolog
TW: TWIST1

## 1 Introduction

Despite intensive research and clinical efforts, grade IV glioblastoma (GBM) remains a highly lethal and treatment‐resistant disease with a median survival time of approximately 12–16 months. Among the major barriers to improved outcomes are the diffusely invasive growth of glioma and the difficulty in eradicating treatment‐resistant glioma stem cells (GSCs). These observations provide strong therapeutic rationale to target mechanisms which coordinately regulate invasion and GSC phenotypes. We and others have reported that TWIST1 (TW), a bHLH transcription factor (TF) and master regulator of epithelial‐to‐mesenchymal transition (EMT), is associated with increased glioma grade and promotes invasion and glioma stem cell (GSC) phenotypes (Elias *et al*., 2005; Mikheeva *et al*., 2010; Rahme and Israel, 2015). While these findings corroborate the well‐characterized functional significance of TW in other cancers, the practical importance of TW as a therapeutic target in GBM has yet to be validated in preclinical GBM models. Consequently, the TW‐regulated molecular pathways that may contribute to GBM tumorigenicity are also not established. This understanding is critical however as a prerequisite to evaluating the potential of therapeutic strategies based on direct inhibition of TW or its downstream regulated pathways.

In carcinomas, TW activates EMT and promotes cancer cell invasion, metastasis, and carcinoma stem cell phenotypes which contribute to treatment resistance and cancer progression (Ramakrishna and Rostomily, 2013) (Sanchez‐Tillo *et al*., 2012). Ectopic expression of TW in transformed mammary cells promotes EMT, stem cell phenotypes, and tumorigenicity (Mani *et al*., 2008), while inhibition of TW decreases metastasis without altering the growth of the primary tumor site (Yang *et al*., 2004). These data support the critical role of TW for carcinoma malignancy and its potential importance as a therapeutic target which has been documented in human nonsmall cell lung carcinoma (NSCLC) xenograft models where TW knockdown inhibits tumor growth (Burns *et al*., 2013; Lv *et al*., 2015; Tran *et al*., 2012). To date, no anti‐TW‐specific drugs have been developed, although doxycycline has some activity as a nonspecific inhibition of TW in lung carcinoma (Qin *et al*., 2015) and TW‐specific RNAi was successfully delivered to breast carcinoma xenografts using a dendrimer (Finlay *et al*., 2015). The lack of available clinically validated therapies to target TW reflects the inherent difficulty of traditional approaches to drugging a TF and underscores the rationale to identify downstream signaling as surrogate targets. Although mTOR was identified downstream of TW in a NSCLC xenograft study, additional actionable surrogate targets for TW have not been identified in human xenograft‐based preclinical models (Lv *et al*., 2015).

In human glioma, an EMT‐like process is thought to regulate malignancy, albeit in the absence of metastasis to distant organs. For instance, gene expression array studies identified a mesenchymal stem cell phenotype in human GBMs (Tso *et al*., 2006) and distinct proneural, proliferative, and mesenchymal gene expression signatures among malignant grade III and IV human gliomas (Phillips *et al*., 2006). The mesenchymal GBM signature is associated with poor prognosis, increased angiogenesis, and tumor recurrence (Morokoff *et al*., 2015), and a proneural‐to‐mesenchymal transition (PMT) commonly accompanies tumor progression and treatment resistance (Segerman *et al*., 2016). As stated above, we and others have reported functional roles of TW in glioma to promote EMT‐associated phenotypes of invasion and cancer cell stemness consistent with those reported in carcinomas (Kubelt *et al*., 2015; Mikheeva *et al*., 2010). However, the potential therapeutic benefits of targeting TW have not been directly tested *in vivo* in human GBM xenograft models. This is a critical first step for establishing therapeutic relevance given that functional phenotypes of TW inhibition as currently understood *in vitro* may not fully translate to a therapeutic benefit *in vivo*.

Therefore, as a first step toward establishing the potential therapeutic benefits of targeting TW in GBM, we studied the effects of TW loss of function on tumorigenicity in xenograft models using the established human U87MG and GBM4 GSC lines. In both cell lines, TW loss of function mitigated glioma cell tumorigenicity with concurrent inhibition of periostin (POSTN) expression and PI3K/AKT signaling. Together, these findings provide evidence for the relevance of TW as a potential therapeutic target in GBM and identify potentially actionable surrogate therapeutic targets.

## 2 Methods

### 2.1 Cell lines

U87MG cells were acquired from ATCC‐ and U87MG‐derived cell lines were maintained in DMEM/F12 with 10% FB Essence (VWR, Radnor, PA, USA). GBM4 was obtained from H. Wakimoto (Wakimoto *et al*., 2009).

### 2.2 CRISPR gene editing, stable, and inducible shRNA knockdown

Two TW targeting gRNA were cloned in pCLIP harboring Cas9 fused to Flag tag (puromycin resistant) and Cas9 targeting gRNA (without Cas9) tagged with RFP (Transomics, Huntsville, AL, USA). Multiple clones were selected for each gRNA and used as a pool. Two independent shRNA for stable (pLKO; Sigma, St. Louis, MO, USA) and conditional knockdown of TW (shTWi in pTRIPZ; Dharmacon, Lafayette, CO, USA) and corresponding vectors harboring scrambled control sequences were used for TW knockdown experiments. For rescue experiments, cells were transduced with retroviruses harboring TW in LXSN or empty vector (Mikheeva *et al*., 2010).

### 2.3 Orthotopic and flank xenograft models

Animal experiments were performed according to approved University of Washington IACUC protocols. Nude mice were injected subcutaneously with 600 000 U87MG cells harboring conditional shTWi or shScri knockdown vector. After 14 days, animals were switched to doxycycline supplemented chow (Envigo, Huntingdon, UK) and sacrificed after 3, 5, or 10 days. Tumor was measured with caliper, and volume was calculated (Mikheev *et al*., 2004). Intracranial injection of cells (100 000–150 000 cells per animal) were performed as described previously using stereotactic frame (Mikheev *et al*., 2015). Peri‐operative care included approved anesthesia and analgesia. Animals were sacrificed when developed clinical manifestation. To avoid animal suffering, the time of sacrifice was used as a surrogate for survival analysis. GFP‐positive tumor fragments identified under fluorescence dissecting microscope were digested with trypsin and plated in media or snap‐frozen for RNA or protein extraction using RNAeasy kit (Qaigen, Hilden, Germany) or RIPA buffer, respectively.

### 2.4 RNAseq analysis

Four biological replicas for each group of cells and eight individual tumors derived from the same cells *in vivo* are included in the study. RNAseq reads were analyzed using FastQC for quality control. Short reads were aligned to the hg38 reference genome. FPKMs (fragments per kilobase per million mapped reads) (Mortazavi *et al*., 2008) were determined for all RefSeq genes using CuffLinks. Read counts were obtained using HTseq (Anders *et al*., 2015). Differential expression analysis was performed based on the read counts, using DESeq2 (Love *et al*., 2014) modeling the raw read counts data to follow a Negative Binomial distribution. The procedure of Benjamini and Hochberg (1995) was used for multiple testing to estimate the FDR. The differentially expressed genes were selected by two criteria, absolute fold change larger than 2 and adjusted *P*‐values (FDR) less than 0.05. RNAseq data are deposited in Gene Expression Omnibus (GEO) with the accession number http://www.ncbi.nlm.nih.gov/geo/query/acc.cgi?acc=GSE106159.

### 2.5 Bioinformatics

Differentially expressed genes were analyzed using GoMiner and Ingenuity pathway analysis for gene categories and canonical pathways. The PI3K/AKT signaling pathway interactions are downloaded from KEGG (Kanehisa and Goto, 2000) and DLRP (Graeber and Eisenberg, 2001) databases. The gene–gene interactions of PI3K/AKT pathway and fold change were visualized using Gephi (Mathieu *et al*., 2009).

### 2.6 Statistical analysis

Median survival of tumor‐bearing animal cohorts was compared by logrank test using Prism 7 (graphpad Software, La Jolla, CA, USA).

### 2.7 Additional methods

Details on cell authentication, RNAseq, immunofluorescence, qRT‐PCR, western blotting, and antibodies are provided in Appendix S1. Methods for histology, invasion, and proliferation assays were previously described (Mikheev *et al*., 2009, 2012, 2015; Mikheeva *et al*., 2010).

## 3 Results

### 3.1 Disruption of TW protein expression by gene editing inhibits U87MG tumorigenicity

To establish the potential therapeutic relevance of TW inhibition in GBM, we first studied the effects of TW protein knockout in the U87MG cell line. U87MG was chosen for the initial experiments based on its high levels of TW expression, rapid and reproducible tumorigenicity, and ease of subcloning required for selection of CRISPR/Cas9‐mediated knockout clones. To confirm the effects of TW loss of function, TW knockdown was also studied in the GBM4 GSC line (see below). Stable selection of TW‐specific gRNA/Cas9 constructs (TW‐A and B) in uncloned U87MG cells resulted in near complete loss of TW protein compared to controls with nontargeting gRNA/Cas9 constructs (dNT) (Fig. S1A). Upon orthotopic implantation, we observed a 29% prolonged survival in animals harboring the U87MG dTW‐A cells compared to dNT control (Fig.S1B). The gross appearance of tumors examined at terminal time points was similar (Fig.S1C), but examination of dTW‐A tumor‐derived cells demonstrated high levels of TW and low Cas9 protein expression compared to U87MG dTW‐A cells before injection (Fig.S1D). The reduction in Cas9 was confirmed by immunostaining of tumor sections (Fig. S1E). These results indicated that a small minority of cells retaining TW expression (and low or absent Cas9) provided a selection advantage for U87MG growth *in vivo* and that the increased survival likely resulted from a smaller number of TW competent cells.

**Figure 1:**
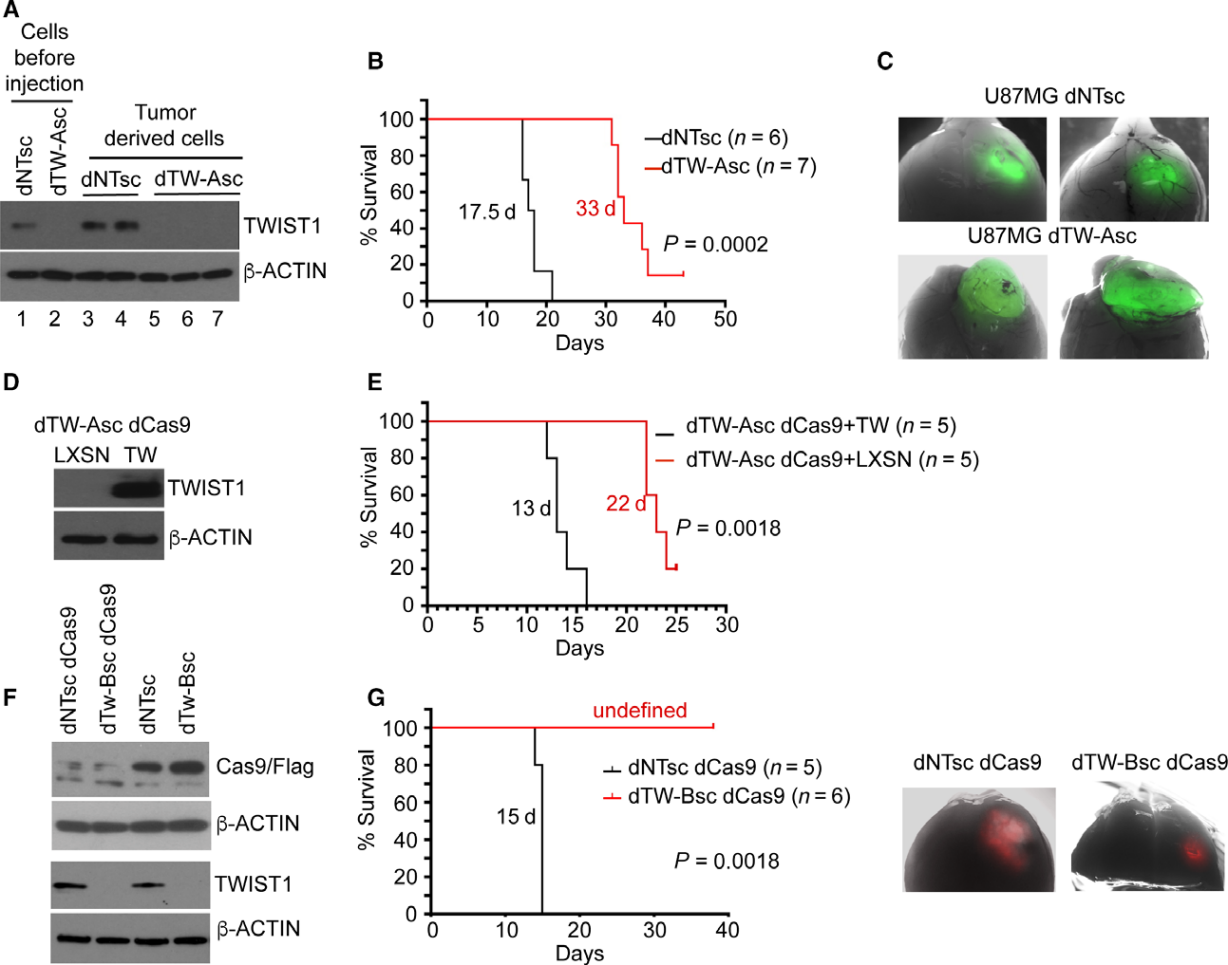
TWIST1 promotes tumorigenicity of U87MG cells. Pooled subclones of TW‐deleted U87MG cells are less tumorigenic: (A) Confirmation of TW expression and knockout retained in tumor‐derived cells from animals injected with control (dNTsc, lanes 3,4) and TW knockout (dTW‐Asc, lanes 5, 6, 7) cells. Cells before injection shown in lanes 1 and 2. (B) Extended survival of animals injected with a pooled subclones with confirmed TW mutation (dTW‐Asc) and knockout compared to pooled control subclones (dNTsc). (C) Differential intracranial versus exophytic growth patterns of tumors generated by U87MG dNTsc and dTW‐Asc cells, respectively. (D) Overexpression of TW or empty vector in U87MG dTW‐Asc cells after self‐deletion of Cas9 (dTW‐Asc dCas9). (E) Survival analysis demonstrating TW re‐expression in TW‐deleted cells significantly reduced median survival compared to TW null control cells. (F) Confirmation of Cas9 and TW knockout in the pooled U87MG subclones expressing the independent TW‐B gRNA (dTW‐Bsc) before injection. (G) Survival curves showing marked prolongation of survival after implantation of TW‐deficient cells (U87MG dTW‐Bsc dCas9) compared to control. (H) Gross appearance of representative TW‐deleted (dTW‐Bsc dCas9) and control tumors (dNTsc dCas9).

To eliminate the outgrowth of rare TW+ cells, we subcloned control U87MG dNT and dTW‐A cells. Mutations and elimination of TW protein were confirmed through direct sequencing across the gRNA site and western blot of selected clones, respectively (Table S1 and Fig. S2). Using algorithm available at KBiobox, we identified two high‐risk off‐target genes for the TW‐A gRNA, USP35, and EXOC4 (Fig. S3). Targeted sequencing found a single mutation only for USP35 in one clone, which was excluded from further experiments (Table S1). Cells were then pooled from 10 random control subclones (designated U87MG dNTsc) and 6 subclones lacking TW expression (U87MG dTW‐Asc) for xenograft experiments (Fig. 1A, lanes 1, 2). After orthotopic implantation of equal numbers of control and TW‐deficient cells, we found that dTW‐Asc implanted animals showed a survival increase of 88% compared to animals implanted with control cells (33 versus 17.5 days, respectively; *P *=* *0.0002; Fig. 1B). Stable loss of TW in U87MG dTW‐Asc‐derived tumors was confirmed in tumor‐derived cells (Fig. 1A, lanes 5‐7 versus 3, 4). Of interest, U87MG dTW‐Asc‐derived tumors generated a partially exophytic versus intracranial growth pattern (Fig. 1C). Histologic examination revealed subtle differences with U87MG dNTsc tumors harboring larger more mesenchymal appearing cells, while U87MG dTW‐Asc‐derived tumors had smaller and more densely packed cells. No tumor cell invasiveness is noted at the tumor brain interfaces independent of TW status (Fig. S4). Re‐expression of TW in dTW‐Asc cells after CRISPR mediated deletion of Cas9 (Fig. 1D) rescued the effects of TW deletion evidenced by a 69% reduction in animal survival compared to U87MG dTw‐Asc cells harboring an empty expression vector (13 versus 22 days, respectively; *P *=* *0.0018) (Fig. 1E) and reversion to exclusive intracranial growth patterns (not shown). With a second gRNA, TW‐B, we validated and pooled six subclones (Table S2; Fig. 1F; no high‐risk off‐target genes were identified) and found a similar decrease in tumorigenicity after implantation (Fig. 1G) and accompanying decreases in tumor growth (Fig. 1H). *In vitro* invasion assay did not reviled effect of TW knockout of cell invasion in serum‐free conditions (not shown). Because U87MG tumors are not invasive *in vivo*, we concluded that inhibition of TW is due to alteration of tumorigenicity and not invasion. Finally, to determine the effect of TW loss of function after tumor formation we showed that doxycycline induced TW knockdown in established U87MG flank tumors reduced subsequent tumor growth from a mean of 300 mm^3^ to 75 mm^3^ (*P *=* *0.0114; Fig. S5). Together, these data strongly support the physiologic relevance of TW for U87MG tumorigenicity and potential relevance of TW as a clinical target.

### 3.2 Identification of TW‐regulated genes and pathways

**Figure 2:**
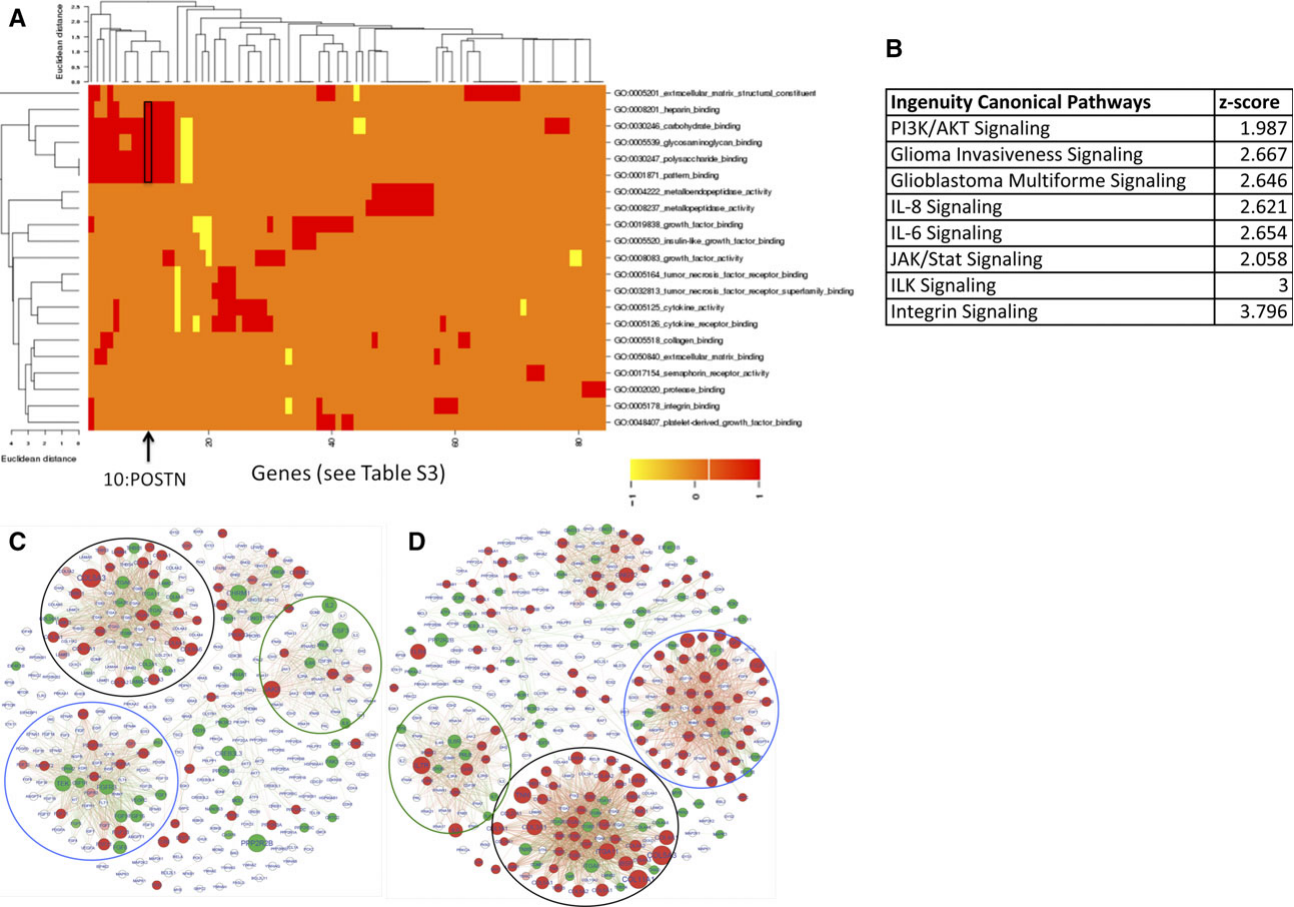
TWIST1 regulates genes associated with ECM and PI3K signaling. (A) Heat map of GO categories of TW DEGs identified by RNAseq in U87MG tumors. GO analysis shows enrichment for categories related to ECM constituents, ECM protein interactions, membrane receptors, growth factor, and cytokine signaling. Genes comprising the significantly altered GO categories (*P *<* *0.05) are listed in Table S4. Scale: Yellow and red show direction of gene expression change (down and up, respectively). POSTN is identified in multiple GO categories (arrow). (B) IPA analysis of DEGs. Examples of canonical pathways identified by IPA analysis and corresponding *Z*‐scores are shown. (C,D) Comparison of *in vitro* (C) and *in vivo* (D) TW‐regulated PI3K network analysis constructed from TW‐dependent DEGs using KEGG. Individual genes (nodes), gene interactions (edges), and proportional absolute log2 fold changes (node size) and direction of change in control versus deleted tumors [upregulation—red (log2 > 0.5‐fold); downregulation—green (log2 < ‐0.5‐fold); no change (white)] are shown. Analogous subnetworks showing the largest changes are circled; growth factor/RTKs (blue), ECM proteins/Integrins (black), and cytokines/cytokine receptors (green). Enlarged version of this panel is shown in Fig. S7.

To identify mechanisms regulated by TW which contribute to tumorigenicity, we performed RNAseq analysis of U87MG dNTsc vs dTW‐Asc cells growing *in vitro* and their respective orthotopic xenograft tumors harvested at terminal time points. A disambiguation analysis was used to eliminate confounding reads from mouse mRNA in xenografts (Ahdesmäki *et al*., 2017). Comparison of TW‐associated differentially expressed genes (DEGs) from cell lines and tumors identified a largely distinct set of DEGs unique to the cell lines or tumors with a smaller number common to both (Fig. S6, Table S3). To identify physiologically relevant candidates, we analyzed all DEGs detected in tumors. GoMiner (Zeeberg *et al*., 2003) analysis identified TW‐regulated DEGs in tumors enriched for genes encoding extracellular space (growth factors, cytokines, extracellular matrix proteins, proteins with metallopeptidase activity) and cell membrane (growth factor receptors, integrins, tumor necrosis factor receptors, and cytokine receptors) proteins (Fig. 2A). This analysis indicated that TW might have functional roles in modifying and/or responding to the *in vivo* tumor microenvironment, cell–cell interactions, ECM‐cell signaling, and response to growth factors. Ingenuity Pathways Analysis (IPA) identified canonical pathways related to glioblastoma signaling and invasiveness indicating that TW has broad effect on U87 glioma cells (Fig. 2B). Among canonical IPA pathways, we identified PI3K/AKT, which is part of the Glioma Invasiveness Signaling and GBM signaling networks. Consistent with GO cytokine categories, we identified IL‐6 and IL‐8 pathways, which signal through JAK/Stat and PI3K/AKT pathways. Alteration of extracellular components is consistent with Integrin and ILK signaling which also signal through PI3K/AKT. (Fig. 2B). Together, GoMiner and IPA suggested that TW likely to promote malignant phenotypes through coordinated regulation of genes related to ECM, membrane receptors, signaling transduction kinases, and cytoskeleton dynamics all of which in part function through PI3K/AKT signaling. TW has been linked to this mechanism (Martini *et al*., 2014), and therefore, we used the Kyoto Encyclopedia of Genes and Genomes (KEGG) database to perform network analysis of PI3K/AKT‐related DEGs. We found that functionally related subsets of PI3K/AKT‐related DEGs were markedly upregulated in TW wild‐type versus TW knockout tumors and that this upregulation was more pronounced in tumor samples compared with cell lines *in vitro* (Fig. 2C, D, Fig. S7). Together, our observations indicate that TW function can be influenced by the environmental context (*in vitro* versus *in vivo*) and that an *in vivo* dependent activation of PI3K/AKT‐related genes may contribute to its impact on tumorigenicity.

### 3.3 TW‐mediated regulation of AKT and periostin

**Figure 3:**
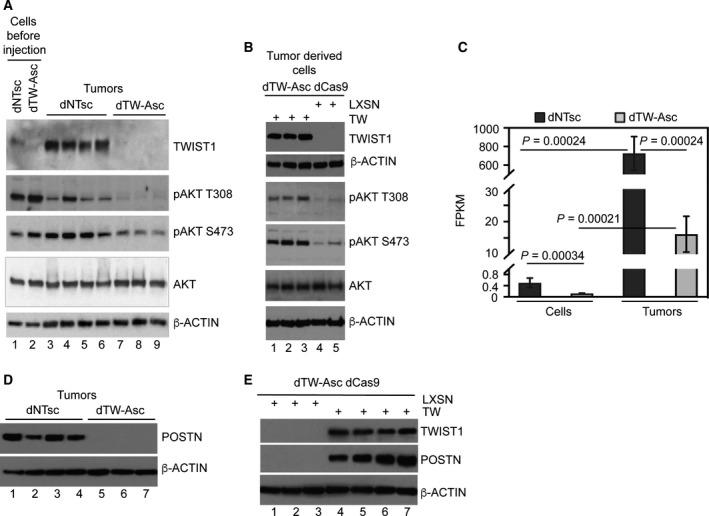
TWIST1 knockout regulates POSTN expression, AKT phosphorylation. (A) Western blot analysis of AKT phosphorylation (T308 and S473) in cells before implant (lanes 1,2) and in individual control (lanes 3–6) and TW‐deleted (lanes 7–9) tumors. (B) Western blot analysis of AKT phosphorylation (T308 and S473) in tumor‐derived early passage U87 dTw‐Asc dCas9 cells re‐expressing TW (lanes 1–3) or with TW deleted (lanes 4,5). (C). *POSTN* mRNA expression (FPKM values from RNAseq) in U87MG cells *in vitro* (cells) and *in vivo* (Tumors) with endogenous (dNTsc) or deleted TW (dTW‐Asc). (D) Western blot detection of POSTN protein in individual control dNTsc (lanes 1–4) and dTW‐Asc tumors (lanes 5–7). Levels of POSTN protein expression in cells *in vitro* are below detection limits (not shown). (E) Analysis of POSTN expression in tumors with deleted TW (dTW‐Asc+LXSN) (lanes 1–3) and in tumors with exogenous TW overexpression (dTW‐Asc + TW) (lanes 4–7).

To establish PI3K/AKT signaling as a downstream target, we investigated TW‐dependent regulation of AKT phosphorylation in U87MG cells and tumors. Marked inhibition of T308 and S473 AKT phosphorylation was observed in tumors but not cell lines prior to implantation (Fig. 3A) and inhibition was reversed in tumor‐derived cells re‐expressing TW (Fig. 3B). Collectively, these data demonstrated that TW‐dependent growth of U87MG cells *in vivo* was associated with activation of AKT. In other cancers, TW regulates AKT signaling through phospho‐activation (Li and Zhou, 2011) (Roberts *et al*., 2016) as well as transcriptional upregulation (Cheng *et al*., 2007). Here, TW deletion did not alter AKT gene expression (not shown), and therefore, in U87MG TW appears to primarily regulate AKT signaling through phosphorylation.

We previously showed that POSTN is important for glioma tumorigenicity (Mikheev *et al*., 2015). POSTN is also known to activate AKT (Morra and Moch, 2011). To determine whether POSTN is a downstream target of TW in the U87MG model, we analyzed POSTN expression in U87MG cell lines and tumors. We found that *POSTN* mRNA expression levels were dramatically increased in U87MG derived tumors compared with cell lines *in vitro* (~1200‐fold; mean FPKM 750 vs 0.4, respectively; *P *=* *0.00024) with a marked ~43‐fold inhibition of *POSTN* expression in TW‐deleted tumors (FPKM 750 vs 17.5; *P *=* *0.00024) also reflected in western blot analysis (Fig. 3C,D). Inhibition of POSTN protein expression in TW‐deleted tumors is reversed by TW overexpression (Fig. 3E). Therefore, as for AKT, POSTN is an *in vivo* target of TW regulated on mRNA level in U87MG.

### 3.4 TW knockdown inhibits GBM4 glioma stem cell tumorigenicity, POSTN expression, and AKT activity

**Figure 4:**
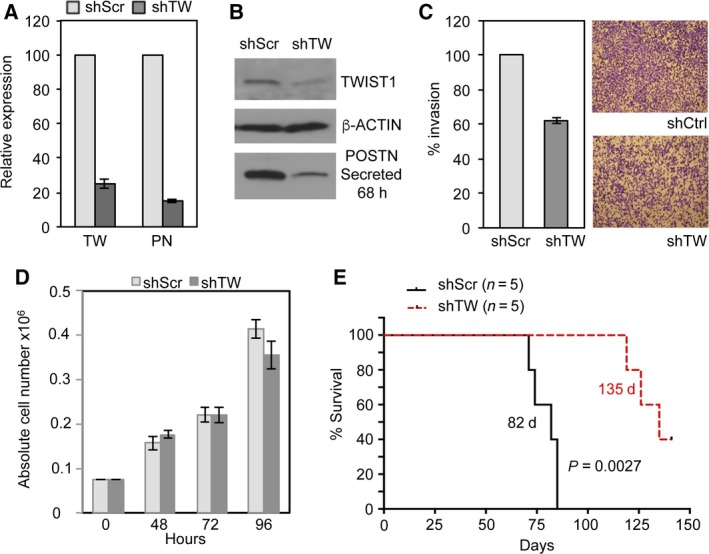
TWIST1 promotes tumorigenicity and upregulates POSTN in GBM4 GSCs. (A) qRT‐PCR quantification of TW and POSTN mRNA levels after TW knockdown in GBM4 with TW‐specific shRNA (shTW) compared to scrambled shRNA control (shScr). (B) Western blot of TW (whole cell lysate) and secreted POSTN from control (shScr) and TW knockdown (shTW) GBM cells. (C) Quantification of matrigel transwell invasion assay with representative membranes shown after 63 h. (D) Proliferation assay of GBM4 shScr vs shTW cells. Absolute cell number was measured at indicated time points. Number of cells plated is shown at time ‘0’. (E) Kaplan‐Meier curves comparing median survival of host nude mice after orthotopic implants of control (shScr) and TW knockdown (shTW) (135 vs 82 days, respectively; *P *=* *0.0027).

**Figure 5:**
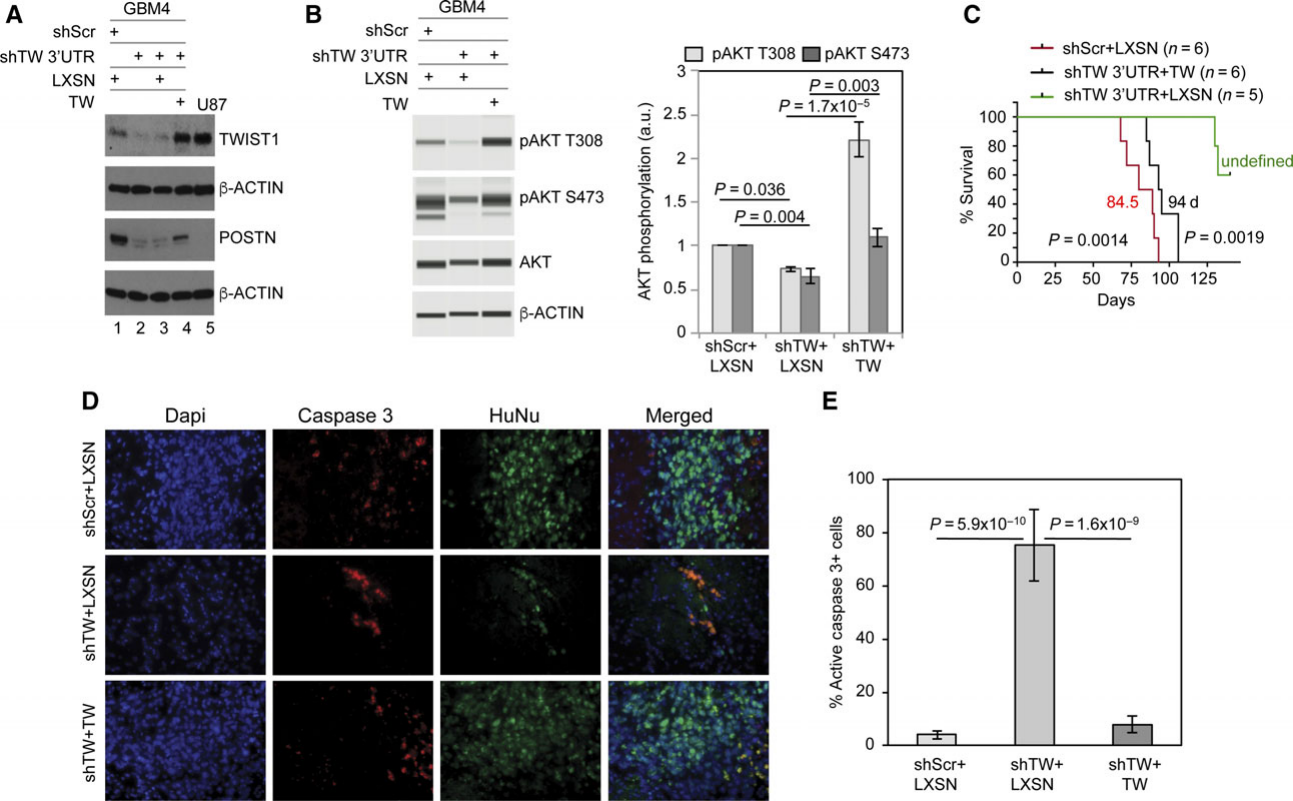
TWIST1‐dependent AKT phosphorylation in GBM4 GSCs correlates with tumorigenicity *in vivo*. (A) Marked knockdown TW and POSTN protein expression in GBM4 cells (whole cell lysates) expressing shScr control or a TW‐specific 3′UTR shRNA (lanes 1,2 respectively). Re‐expression of TW open reading frame TW partially restores POSTN expression compared to empty vector (lanes 3, 4). U87MG is shown for comparison of TW expression and lack of POSTN expression (lane 5). (B) TW knockdown in GBM4 reduced AKT phosphorylation (T308 and S473). Overexpression of TW increases AKT phosphorylation. (C) 3′ UTR‐mediated TW knockdown increased survival (>125 days versus 84.5 days; *P *=* *0.0014), while re‐expression of TW restores the control tumorigenic phenotype (median survival 94 days). (D) Representative images of tissue sections from short‐term (3‐4 animals/group) implants of control (top row), TW 3′UTR knockdown (middle row), and TW re‐expression (bottom row) in GBM4 cells. Animals were sacrificed 10 days after intracranial cell injection. Staining was performed for total nuclei (DAPI), apoptotic cells (active caspase 3), and tumor cells (HuNu). (E). Quantification of active caspase‐positive cells in implants of control cells (shScr+LXSN), cells with TW knockdown (shTW+LXSN), and TW‐overexpressing cells (shTW+TW). Percent of active caspase‐3‐positive cells is calculated relative total number of HuNu‐positive cells in the implant. *P* value calculated by two‐tailed *T*‐test.

To extend our observations in U87MG to a GSC line, we studied the effects of TW knockdown in the GBM4 GSC line. Expression of TW shRNA (shTW) achieved significant knockdown of TW and POSTN mRNA (~75% and ~85%, respectively) and protein levels (Fig. 4A,B) and 40% reduction in invasion (Fig. 4C). TW silencing did not affect cell proliferation in vitro (Fig. 4D), suggesting that differences in cell invasion are not influenced by alteration of cell proliferation. We observed marked improvement in median survival of animals injected with GBM4 shTW cells compared to control (135 days versus 82 days, respectively; *P *=* *0.0027, Fig. 4E). A second TW shRNA targeting the 3′UTR also inhibited POSTN expression, which was partially re‐established by re‐expression of TW (Fig. 5A). Knockdown of TW and corresponding inhibition of POSTN also resulted in reduced AKT phosphorylation, which was restored with re‐expression of TW (Fig. 5B). Bar diagram demonstrates significant averaged change in AKT phosphorylation at Thr308 and Ser473. Expression of the 3′UTR shTW in GBM4 cells markedly prolonged survival (undefined at 132 days versus 84.5 days for controls; *P *=* *0.0014), while TW re‐expression restored tumorigenicity to levels comparable to controls (median survival 94 days) (Fig. 5C). To examine effects of TW early after cell implantation, we examined expression of activated caspase 3 at 10 days postimplantation. TW knockdown decreased the number of viable cells with corresponding increases in activated caspase 3 +  tumor cells compared to controls (Fig. 5D; middle and top panel, respectively). Re‐expression of TW completely reversed this effect (Fig. 5D; lower panel, Fig. 5E). These data demonstrated that TW loss of function in a second GBM cell line resulted in a similar decrease in tumorigenicity, POSTN expression, and AKT signaling as observed above for U87MG.

### 3.5 TW expression correlates with GBM patient survival and POSTN expression

**Figure 6:**
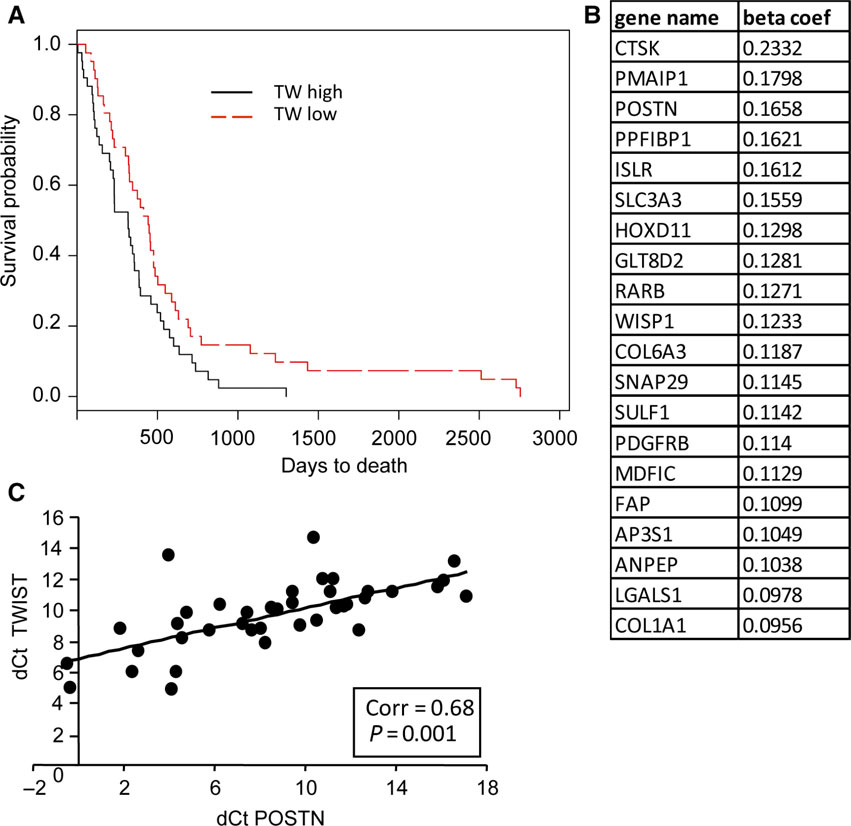
TWIST1 expression levels predict survival and correlate with POSTN expression. (A) Kaplan–Meier analysis of patient survival stratified by TW high and low expression levels (*n *=* *53 each group). (B) List of top ranking genes in TW transcriptional network analysis of TCGA TW DEGs identifies *POSTN*. (C) Correlation between *TW* and *POSTN* expression in a group of 39 glial tumors (9 grade II, 3 grade III, and 27 grade IV). ΔCt values were determined by qRT‐PCR.

To establish potential clinical relevance of TW‐mediated regulation of POSTN, we investigated the relationship between TW expression and GBM patient survival using the TCGA database. TW expression levels distribution across 525 samples in the microarray dataset and in samples with top 10% and bottom 10% levels of TW expression (Fig. S8A, B) were used for survival analysis. We selected to use patient top and bottom TW expressers as it mirrors overexpression and under‐expression in the animal experiments. The array counts generated by TCGA were used without additional processing. Patient median survival was significantly decreased in patients with high (top 10%) versus low (bottom 10%) TW expression levels (Fig. 6A; 318 vs 442 days, respectively; *P *=* *0.0331). Similar results were observed in the TCGA RNAseq dataset (Fig. S8C). Similarly by comparing high TW‐expressing GBMs with all other GBMs in the TCGA data set based on a *Z*‐score cutoff of 2 (http://www.cbioportal.org), we found a comparable level of significance (340 vs 435 days, *P *=* *0.0307, *n *=* *487; data not shown). A similar impact of high TW expression on shortened survival was reported using REMBRANDT data comprised of all glioma grades (Nordfors *et al*., 2015) and in NSCLC (Lv *et al*., 2015). These observations suggest that high levels of TW expression have a generic effect on increasing cancer malignancy.

To establish potential mechanistic links between TW and POSTN in GBM, we generated a TW transcriptional regulation model for all protein‐coding genes using TCGA microarray data as input (Ament *et al*., 2016). By only considering transcription factors for which footprints are found in ENCODE DHS data, we performed LASSO regression to identify the top targets of TW. POSTN was found to be a likely target for TW, having the third best beta‐coefficient among all putative TW targets (Fig. 6B). Further, a significant correlation between TW and POSTN expression levels was detected in a set of 38 human gliomas of various grades (Fig. 6C) supporting potential regulatory interactions between the two genes. Similar significant correlations were observed in Rembrandt and TCGA datasets (*r *=* *0.531, *P *=* *0.00001 and *r *=* *0.414, *P *=* *0.00001; respectively) (Table S5). In the TCGA dataset (U133A microarrays; *n *=* *358), we also determined that the significant correlation between TW and POSTN is driven largely by high correlations in the proneural (*R *=* *0.5; *P *=* *0.000075; *n *=* *57) and mesenchymal (*R *=* *0.469; *P *=* *0.0002, *n *=* *58) but not neural or classical GBM subtypes (Table S5). Together, these results demonstrated that high TW expression is associated with increased GBM malignancy and POSTN expression supporting the broader potential clinical relevance of TW‐mediated regulation of POSTN for malignancy and that targeting this signaling axis may be most productive in specific GBM subtypes.

## 4 Discussion

TW is a well‐established master regulator of EMT in carcinoma (Qin *et al*., 2012; Zhao *et al*., 2017; Zhu *et al*., 2016), and loss of TW function has been shown to decrease tumorigenicity in lung carcinoma xenografts (Burns *et al*., 2013; Lv *et al*., 2015; Tran *et al*., 2012), but existing data on TW function in glioma is restricted to *in vitro* functional analysis or correlations of expression in human tissue samples with clinical features of malignancy or outcome (Elias *et al*., 2005; Mikheeva *et al*., 2010; Nordfors *et al*., 2015). The purposes of this study were to demonstrate the potential therapeutic relevance of targeting TW in human GBM and identify mechanisms downstream of TW. To establish the potential therapeutic relevance of TW, we tested the impact of TW loss of function in human GBM cell lines on tumorigenicity and employed transcriptomic analysis to identify TW‐regulated signaling mechanisms. Also, we identified downstream TW signaling mechanisms, which might serve as surrogate targets to inhibit function. Our results provided the first *in vivo* evidence supporting the clinical relevance of targeting TW in GBM and confirmed POSTN and AKT signaling as candidate effectors of TW function in these models. Importantly, we also validated the clinical relevance of POSTN as a putative TW target gene in human glioma and GBM gene expression databases.

Using either CRIPSR/Cas9 gene editing or shRNA knockdowns in two GBM cell lines, we observed consistent decreases in tumorigenicity and selection of TW‐expressing cells in uncloned cell pools (Fig. S1). Of interest, the elimination of Cas9 resulted in a slight increase in tumorigenicity (Fig. 1B vs Fig. 1E). This may reflect an inhibitory effect of constitutive Cas9 on tumorigenicity through its recognized toxic or off‐target effects (Morgens *et al*., 2017) or differences in malignant potential of clones selected in the different experiments. Regardless, the consistent impact of TW loss of function and rescue on tumorigenicity with TW re‐expression supports its critical physiologic role for U87MG malignancy. TW loss of function also decreased tumorigenicity in the GBM4 GSC line, suggesting that TW functions may be critical for malignancy in clinically relevant GSCs. Overall, the consistent results observed in both cell lines employing rigorous controls for nonspecific effects provide strong preliminary evidence for the potential therapeutic relevance of inhibiting TW in GBM. To validate our current findings and more closely simulate the clinical setting, future studies will employ conditional inhibition of TW in established orthotopic GSC xenografts.

Of the few studies reporting on the effects of direct TW inhibition in human cancer xenograft models, all involve the use of human NSCLC lines (Burns *et al*., 2013; Lv *et al*., 2015; Tran *et al*., 2012). Lv *et al*. (2015) and Tran *et al*. (2012) using shRNA‐mediated TW knockdown in single cell lines report decreased growth rate and tumorigenicity, respectively, in flank models (Lv *et al*., 2015; Tran *et al*., 2012). Using five NSCL lines, Burns *et al*. (2013) showed that shRNA‐mediated inhibition of TW uniformly decreased tumor growth rates through activation of oncogene‐induced senescence independent of specific oncogene drivers or p53 mutation status. The cell lines used in the present study also harbor different driver mutations (PTEN for U87MG and MYC amplification for GBM4) and p53 mutations status (U87MG WT and GBM4 mutant (not shown)) yet demonstrate similar reduction in tumorigenicity with TW loss of function. TW can interact with WT p53 and promote its degradation (Piccinin *et al*., 2012) indicating potential unique functional significance in p53 WT tumors. However, our previous study (Mikheev *et al*., 2017), the present study, and others (Burns *et al*., 2013) demonstrate that TW can support tumorigenicity independent of p53 mutational status and as shown in a mouse model of skin cancer (Beck *et al*., 2015), TW may have p53‐dependent and p53‐independent functions that drive malignancy. These reports and our observations here provide rationale for additional studies in GBM to establish the broader scope of oncogenic contexts in which TW may be a therapeutic target.

The downstream effects of TW inhibition have not been rigorously characterized in human xenograft glioma models. Global analyses of TW loss of function in a cranial mesoderm development model identified cell–matrix interaction, regulation of EMT, blood vessel morphogenesis, and anti‐apoptosis via PI3K/AKT as the top four enriched processes (Bildsoe *et al*., 2016), which strikingly mirror the findings of our transcriptome analysis of TW knockout in U87MG xenografts. Informed by this analysis, we also validated an association between TW‐dependent tumorigenicity and regulation of AKT signaling and POSTN expression. Because PTEN mutation is recognized as leading event in activation of AKT signaling in U87MG cells, our results show for the first time that TW knockout is sufficient to interrupt effect of PTEN mutation.

Gene expression profiles of U87MG cells were markedly influenced by *in vivo* growth and revealed TW‐mediated regulation of POSTN and AKT signaling not evident in cells prior to implantation. This observation underscores the importance of studying gene function in the more relevant *in vivo* physiologic context as recently shown in a screen for cancer drivers in GSC models (Miller *et al*., 2017). A similar analysis of GBM4 xenografts was practically limited by the invariable growth and small tumor sizes of TW‐deficient GBM4 tumors, yet these cells demonstrated TW‐dependent regulation of POSTN and AKT without *in vivo* selection. This may indicate that specific oncogenic signaling activity may be altered by *in vivo* selection in a cell type‐specific fashion, which can be established with further investigation.

The functional relevance of POSTN for TW function is suggested by similar inhibition of GBM4 cell survival *in vivo* observed in prior analysis of POSTN (Mikheev *et al*., 2015) and TW loss of function shown here. Our analysis of human GBM TCGA data provided evidence that POSTN is regulated by TW in human glioma, supporting the clinical relevance of findings in our xenograft studies. How TW might regulate POSTN expression in our models requires further investigation but may involve mechanisms identified in other biological models. For instance, Oshima *et al*. demonstrated binding of TW to an E‐box site within the mouse POSTN promoter (Oshima *et al*., 2002) and Connerney *et al*. demonstrated that POSTN expression is highly dependent on the TW homo‐ or heterodimerization motifs (Connerney *et al*., 2006). In GBM and other cancers, POSTN signals through integrin receptors and in other cancers can activate AKT (Bao *et al*., 2004; Mikheev *et al*., 2015; Ruan *et al*., 2009). Of interest, AKT‐dependent phosphorylation of TW at Ser42 is reported to inhibit p53 activity (Vichalkovski *et al*., 2010), suggesting reciprocal interactions between TW and AKT may contribute to malignancy through additional mechanisms of particular relevance in p53 wild‐type GBM cells such as U87.

Together, these observations support a putative TW‐POSTN‐AKT signaling axis in human GBM, which could potentially be targeted to disrupt TW‐mediated tumorigenicity. We previously demonstrated that POSTN loss of function phenocopies TW loss of function as shown here (Mikheev *et al*., 2015). In addition, in a Cre‐Lox TW mouse glioma model we found that TW deletion inhibited tumorigenicity when neural progenitor cells (NPCs) were transformed with Ha‐RASV12 overexpression and p53 knockdown but not with co‐overexpression of Ha‐RASV12 and myristoylated AKT (a constitutively phosphorylated form) (Mikheev *et al*., 2017). The latter finding suggested that TW acts upstream of AKT accounting for the lack of effect when transforming NPCs with the constitutively activated myrAKT (Zhao *et al*., 2003). This is consistent with our current findings here that TW loss of function inhibits the activation of endogenous AKT in human GBM cells, suggesting that TW acts upstream of AKT. Of interest, TW loss of function resulted in altered AKT activation regardless of PTEN status (U87 is PTEN deficient and GBM4 is PTEN wild‐type). This further implies that TW may function downstream of PTEN through mechanisms such as mTORC2 or PDK1 which proximately regulate AKT phosphorylation (Manning and Toker, 2017). Another open question is whether TW regulates AKT and malignancy in GBM through specific AKT isoforms as has been shown for TW‐driven EMT by promoting TW degradation in breast cancer cells (Li *et al*., 2016). Taken together, our current findings provide support for future studies that more precisely dissect the signaling interactions between TW, POSTN, and AKT and establish points of regulation expected to provide mechanistic insight and potential translational relevance for targeting TW‐driven GBM malignancy.

## 5 Conclusions

In this study, we took a critical step toward establishing the potential of TW as a therapeutic target in GBM by documenting inhibition of tumorigenicity with TW loss of function in human xenograft models. In addition, we identified POSTN and AKT signaling as mechanisms downstream of TW, which may expand the therapeutic options to indirectly abrogate TW function. The use of dendrimer delivery of a TW‐specific RNAi has shown theoretical potential in a breast carcinoma model (Finlay *et al*., 2015). Recently, TW inhibitor that phenocopied genetic loss of TW by inducing oncogene‐induced senescence or apoptosis was described (Yochum *et al*., 2017). Strategies to target the POSTN/AKT axis downstream of TW could utilize the following approaches alone or in combination: Delivery of POSTN blocking antibodies demonstrated to have activity in preclinical cancer models (Zhu *et al*., 2011), blockade of POSTN binding integrin receptors with cilengitide (Mikheev *et al*., 2015), and available AKT inhibitors.

## Author contributions

AMM and RCR designed and directed this study and provided conceptual guidance and data interpretation. AMM and SAM served as main contributors, collected, and assembled the data. LJS and JS contributed to data collection. CCF and NDP performed TCGA data analysis and interpretation. JLM‐F and CT performed RNAseq analysis and interpretation. LH and SW performed bioinformatics analysis of RNAseq data. The manuscript was written by RCR and AMM and was commented on by all authors. All authors read and approved the final manuscript.

## Supporting information


**Fig. S1.** Growth advantage of TW‐expressing cells *in vivo*.

**Fig. S2.** (A) A‐insertion generated by dTW‐A targeting gRNA in TW confirmed by single cell subcloning and sequencing. (B) Confirmation of TW protein loss in selected U87 subclones by western blot.

**Fig. S3.** Analysis of dTW‐A target gRNA sequence for off‐target effect.

**Fig. S4.** Representative histologic appearance of terminal tumors derived from U87MG dNTsc and dTW‐Asc (H&E staining). (scale bar = 50 μm).

**Fig. S5** Inducible knockdown of TW in established tumors inhibits subsequent tumor growth.

**Fig. S6** Differentially expressed genes regulated by TW in U87MG cells *in vivo* and *in vitro*.

**Fig. S7.** Enlarged representation of Fig 2C, D.

**Fig. S8**. TCGA Level 3 data (given in array counts) was analyzed with respect to TW expression.

**Table S1.** Summary of single cell clones derived from U87MG cells and verified for mutations in TW and off‐target high‐risk genes: EXOC4 and USP35

**Table S2.** Summary of single cell clones derived from U87MG cells and verified for mutations in TWIST1

**Table S3.** List of differentially expressed genes regulated by TW in U87MG cells *in vivo* and *in vitro*.

**Table S4.** List of genes in Gene ontology categories regulated by tumor microenvironment and by TW status.

**Table S5.** Summary of TW and POSTN expression correlation analyses in TCGA and RMBRANDT data sets.

**Appendix S1.** Supplemental methods.
