# Supplementary figures and images for: Targeting TWIST1 through loss of function inhibits tumorigenicity of human glioblastoma
Source: Mol Oncol. 2018 May 29;12(7):1188–202. doi: 10.1002/1878-0261.12320 (PMC6026950; doi:10.1002/1878-0261.12320)

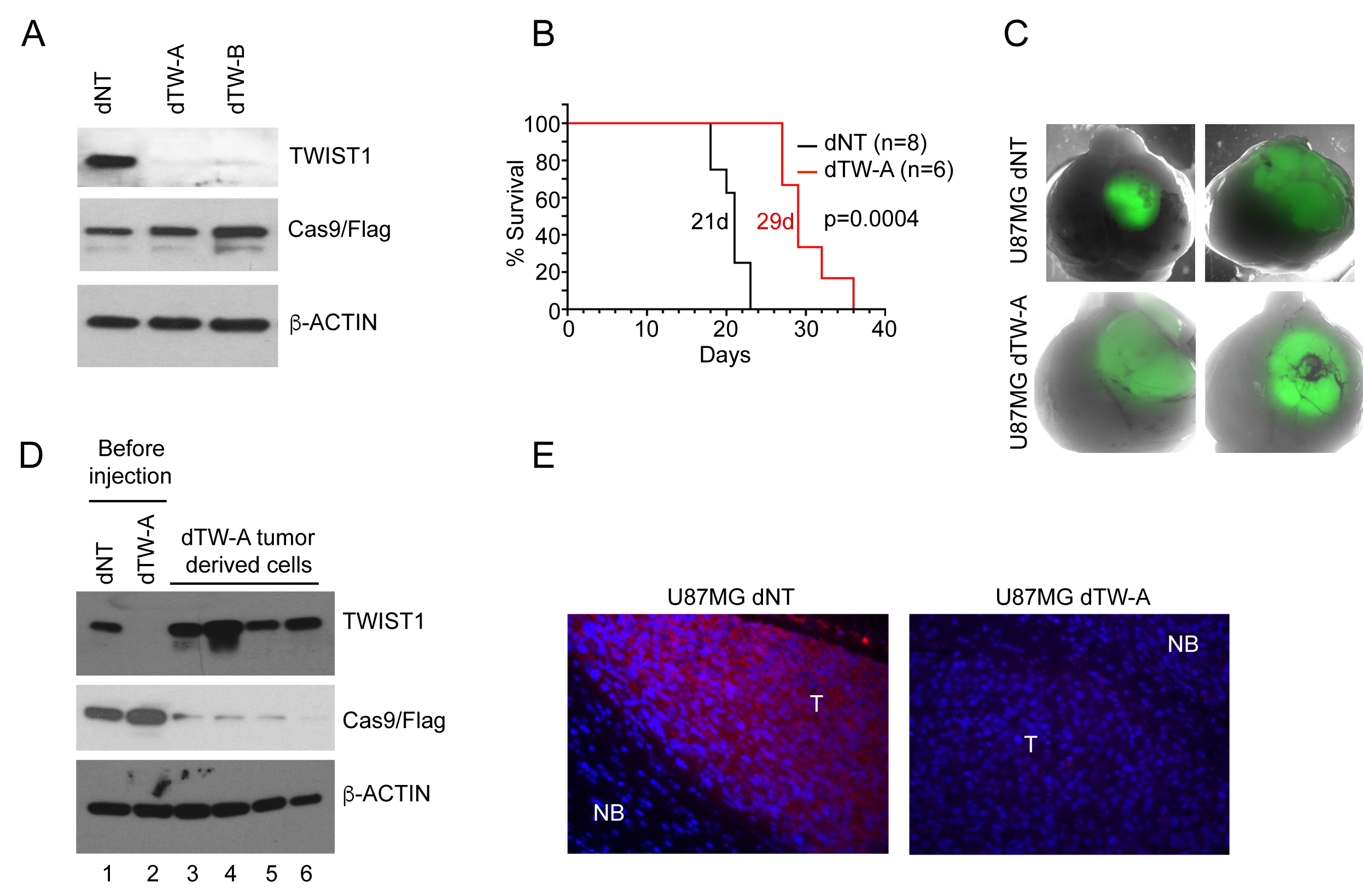

Supplement: Supplementary file 1 — Fig. S1. Growth advantage of TW‐expressing cells in vivo. [file MOL2-12-1188-s001.tif]

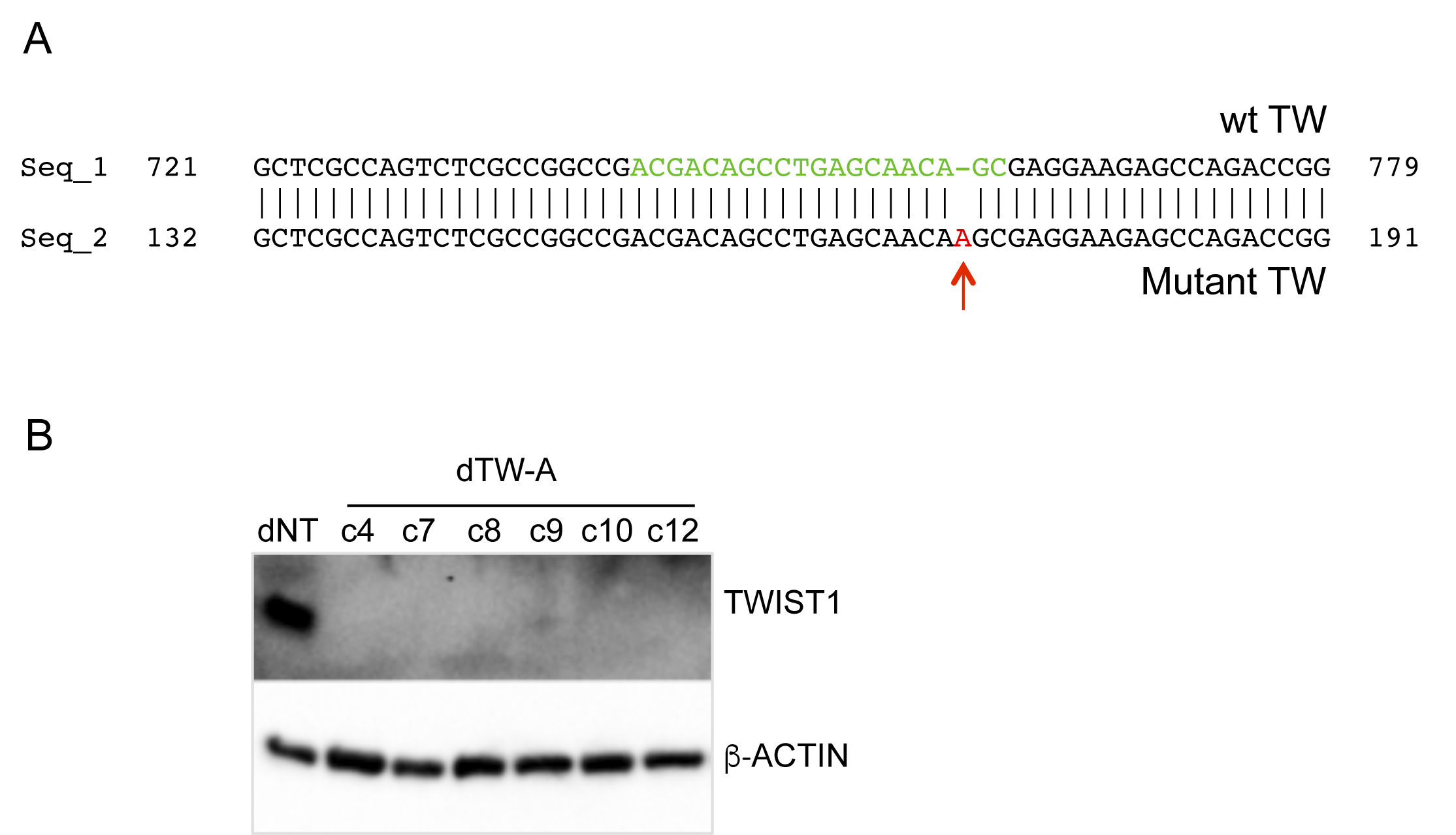

Supplement: Supplementary file 2 — Fig. S2. (A) A‐insertion generated by dTW‐A targeting gRNA in TW confirmed by single cell subcloning and sequencing. (B) Confirmation of TW protein loss in selected U87 subclones by western blot. [file MOL2-12-1188-s002.tif]

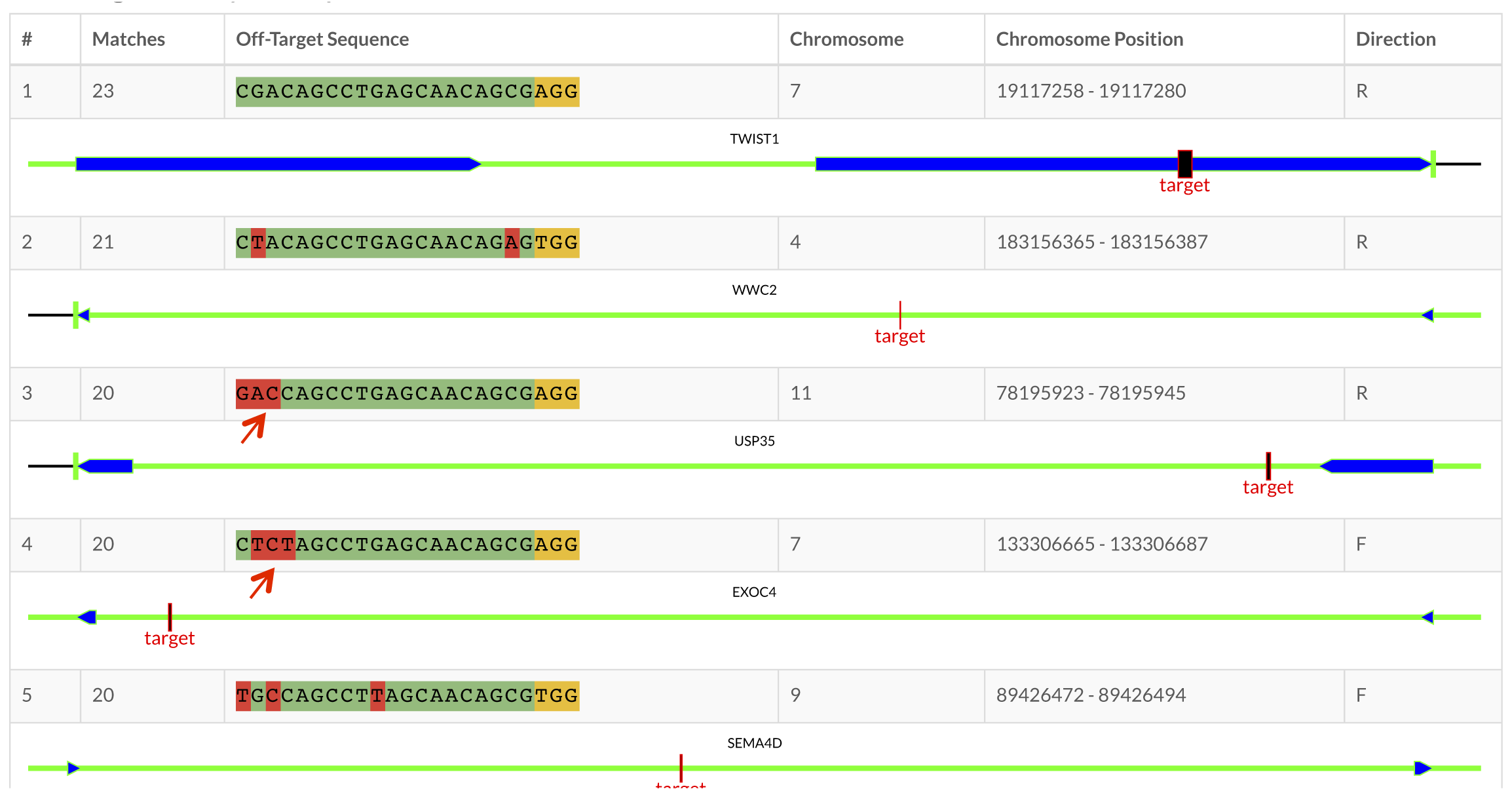

Supplement: Supplementary file 3 — Fig. S3. Analysis of dTW‐A target gRNA sequence for off‐target effect. [file MOL2-12-1188-s003.tif]

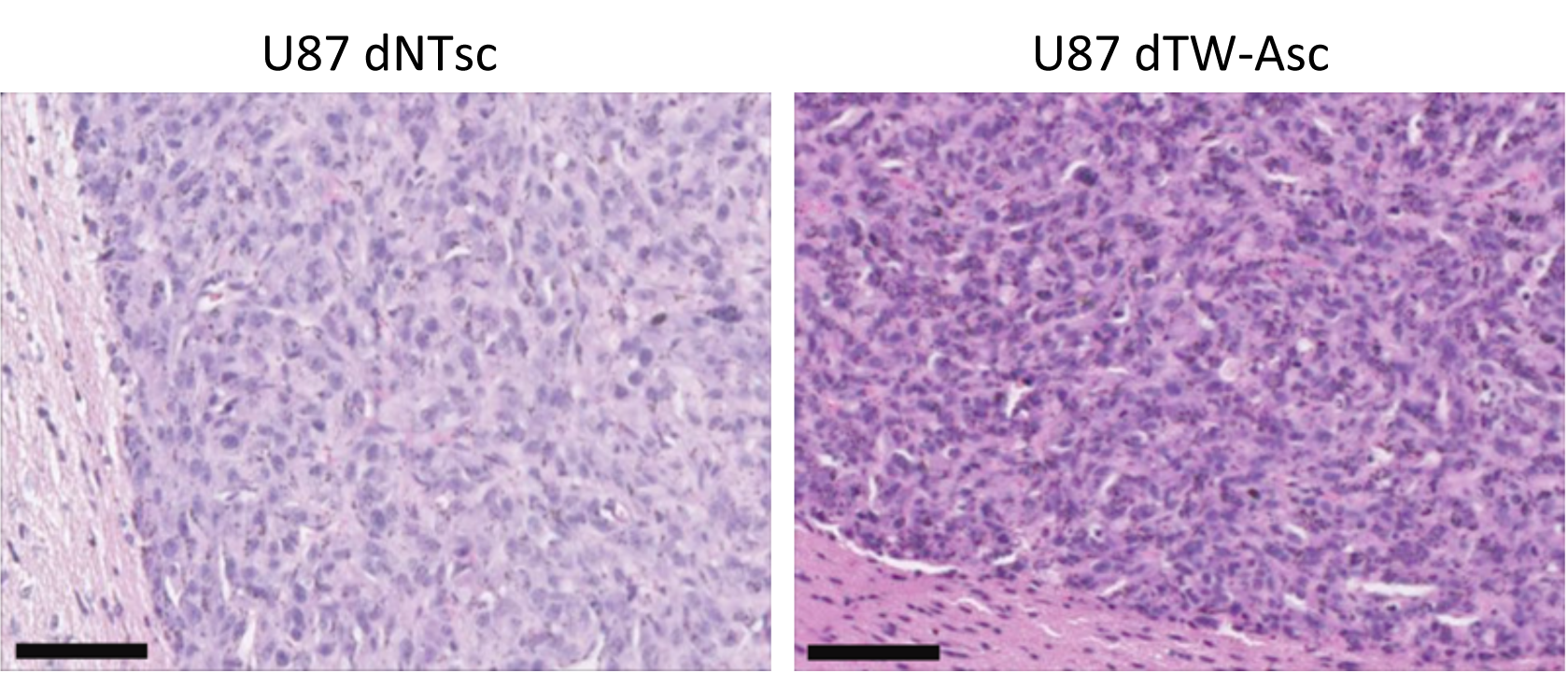

Supplement: Supplementary file 4 — Fig. S4. Representative histologic appearance of terminal tumors derived from U87MG dNTsc and dTW‐Asc (H&E staining). (scale bar = 50 μm). [file MOL2-12-1188-s004.tif]

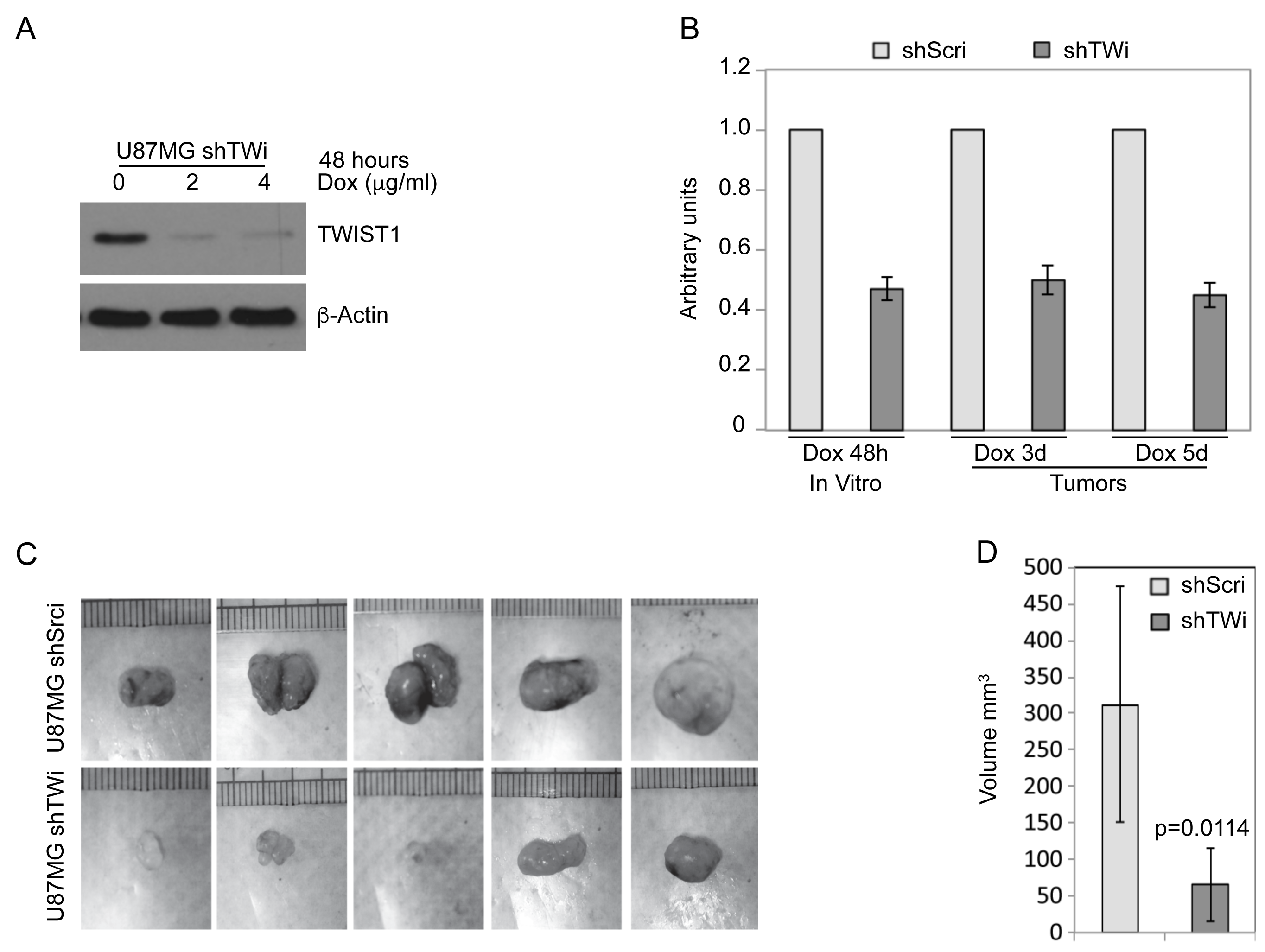

Supplement: Supplementary file 5 — Fig. S5 Inducible knockdown of TW in established tumors inhibits subsequent tumor growth. [file MOL2-12-1188-s005.tif]

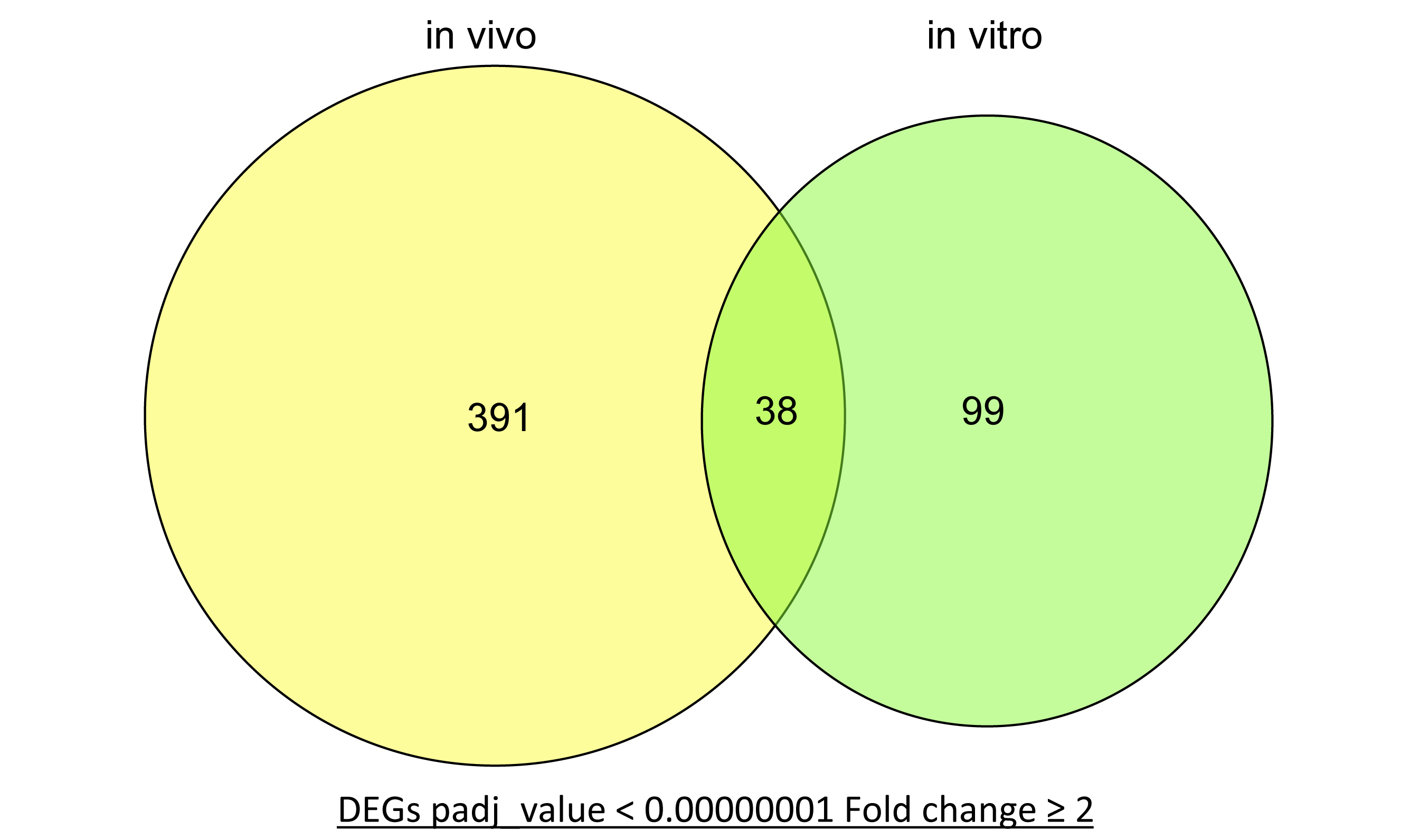

Supplement: Supplementary file 6 — Fig. S6 Differentially expressed genes regulated by TW in U87MG cells in vivo and in vitro. [file MOL2-12-1188-s006.tif]

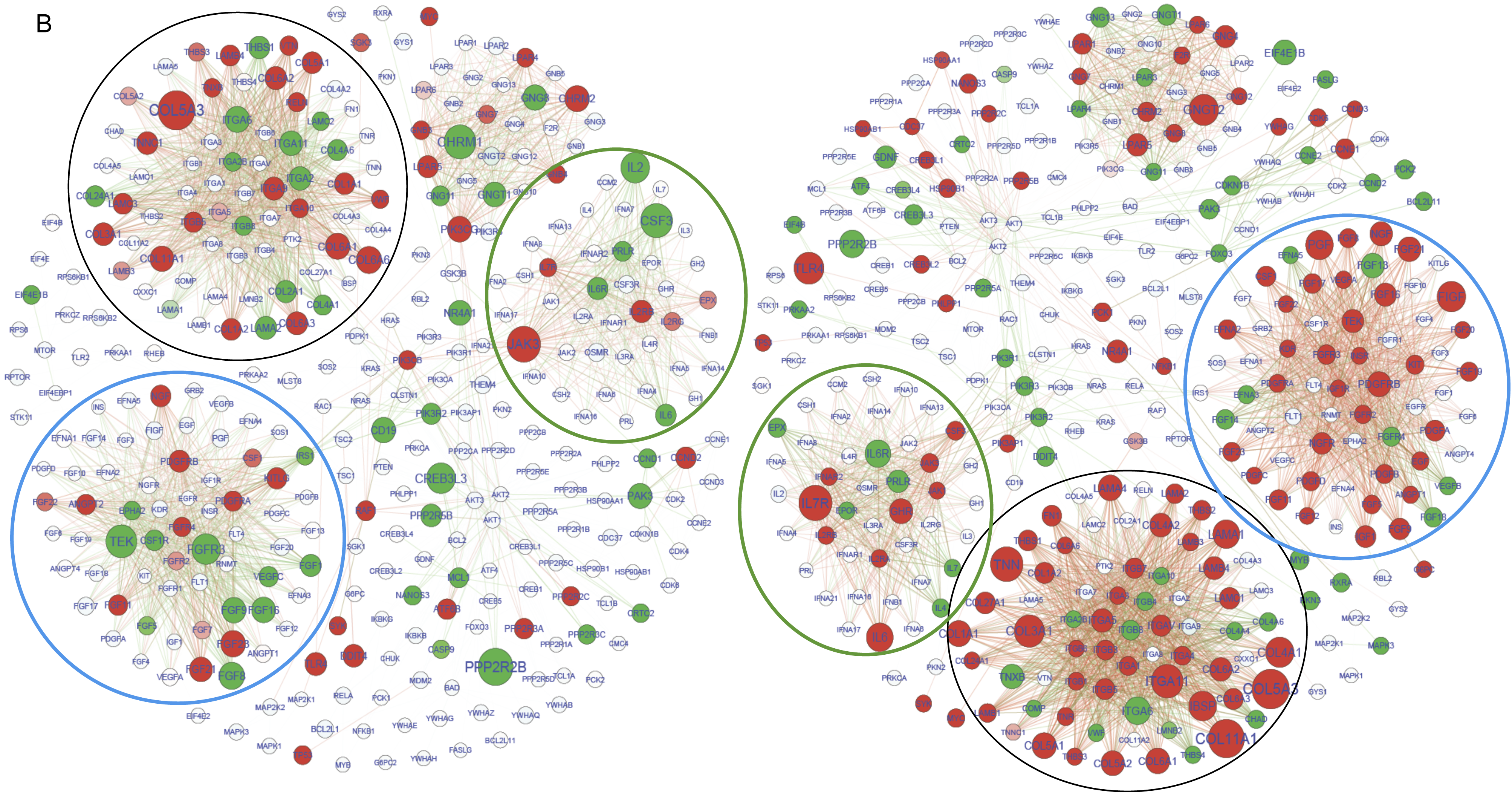

Supplement: Supplementary file 7 — Fig. S7. Enlarged representation of Fig 2C, D. [file MOL2-12-1188-s007.zip › Fig.S7_.tif]

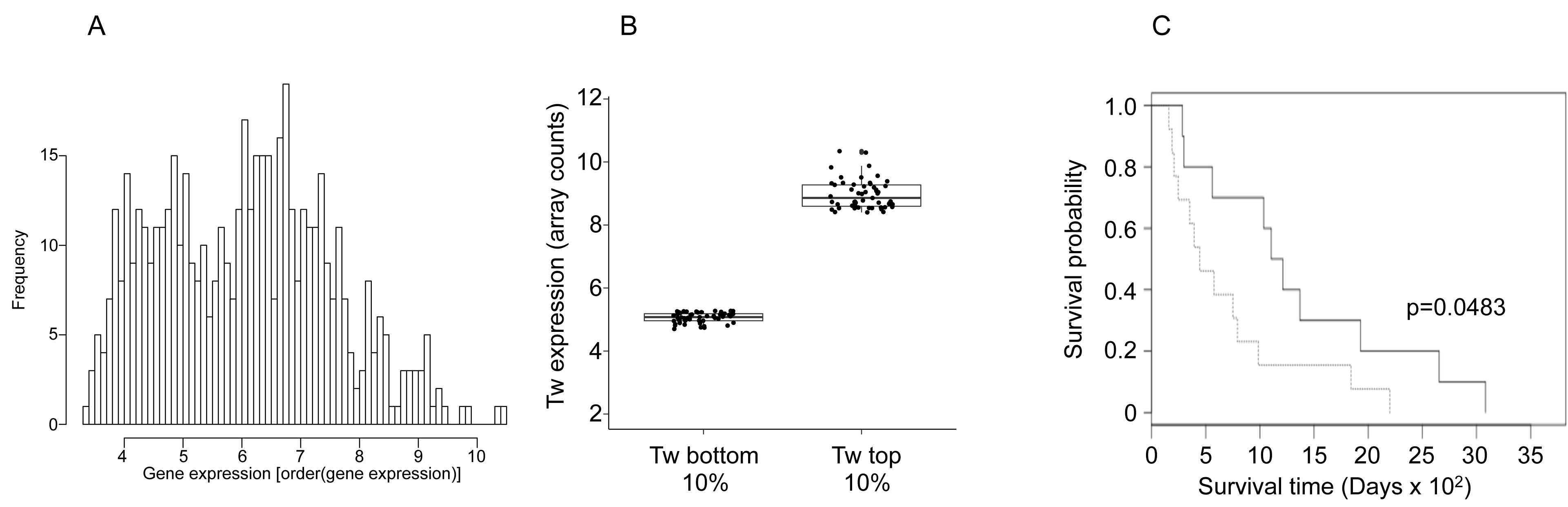

Supplement: Supplementary file 8 — Fig. S8. TCGA Level 3 data (given in array counts) was analyzed with respect to TW expression. [file MOL2-12-1188-s008.tif]

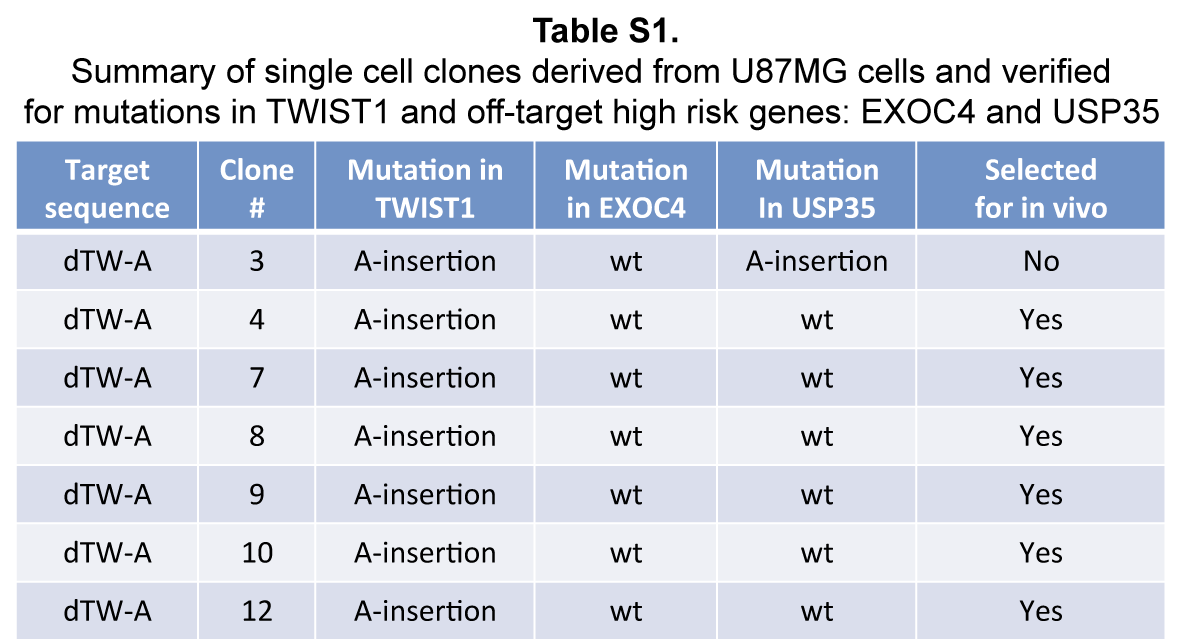

Supplement: Supplementary file 9 — Table S1. Summary of single cell clones derived from U87MG cells and verified for mutations in TW and off‐target high‐risk genes: EXOC4 and USP35 [file MOL2-12-1188-s009.tif]

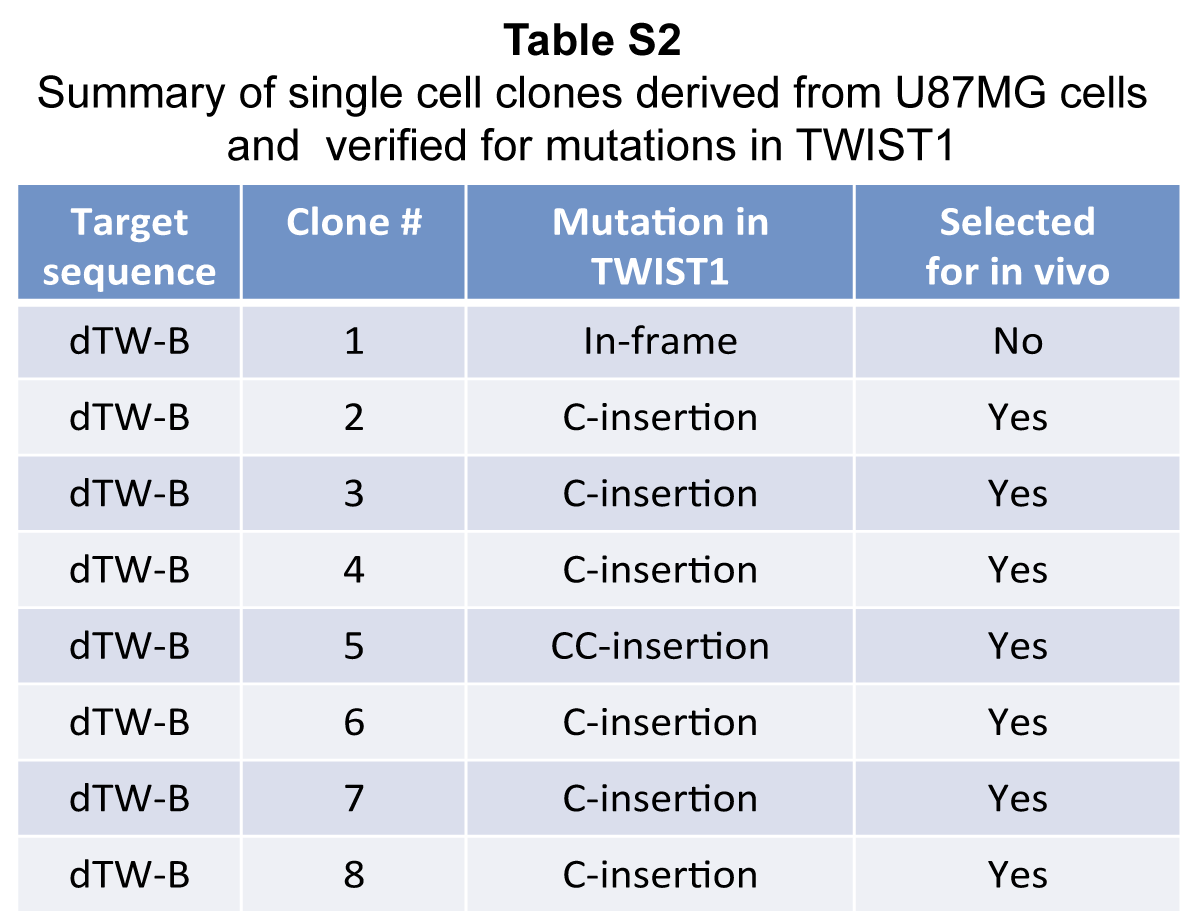

Supplement: Supplementary file 10 — Table S2. Summary of single cell clones derived from U87MG cells and verified for mutations in TWIST1 [file MOL2-12-1188-s010.tif]

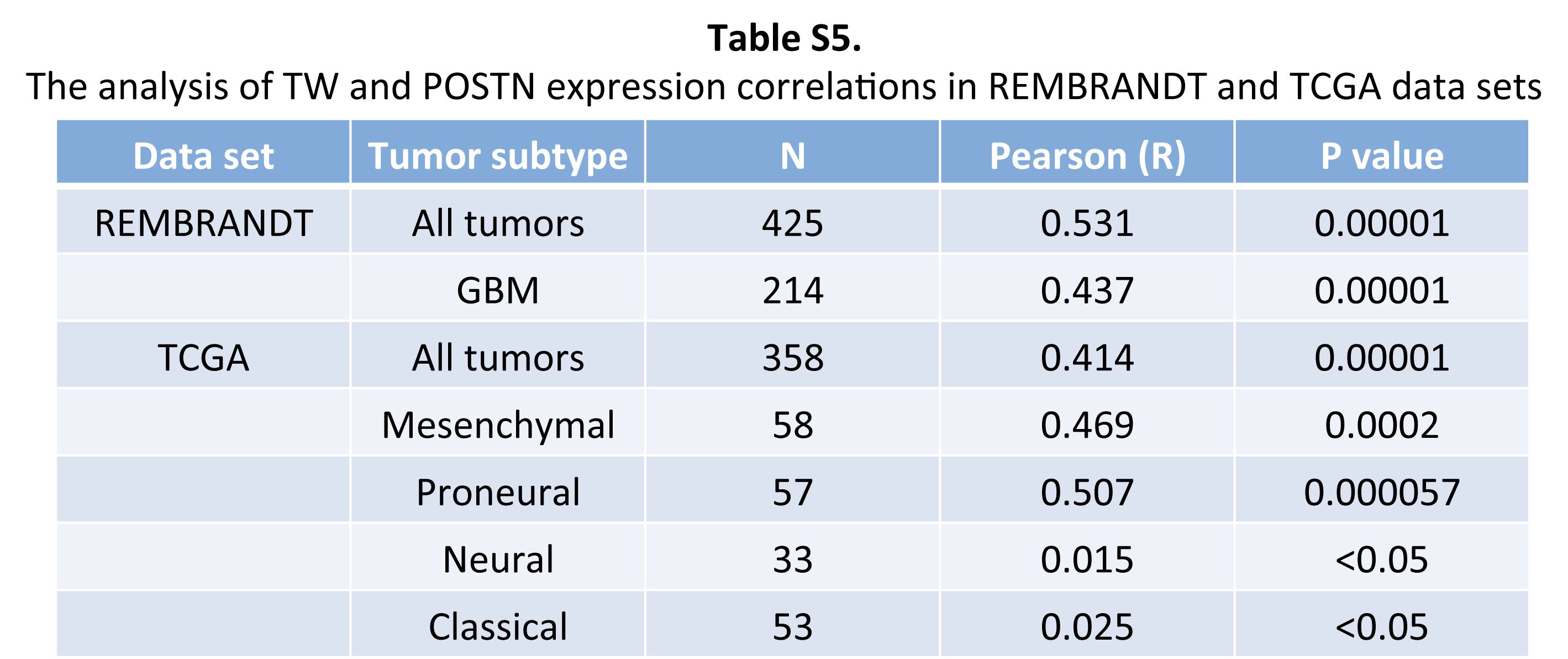

Supplement: Supplementary file 13 — Table S5. Summary of TW and POSTN expression correlation analyses in TCGA and RMBRANDT data sets. [file MOL2-12-1188-s013.tif]
