# Supplementary material for: Targeting TWIST1 through loss of function inhibits tumorigenicity of human glioblastoma
Source: Mol Oncol. 2018 May 29;12(7):1188–202. doi: 10.1002/1878-0261.12320 (PMC6026950; doi:10.1002/1878-0261.12320)
Supplement: Supplementary file 14 — Appendix S1. Supplemental methods. [file MOL2-12-1188-s014.docx]

**SUPPLEMENTAL METHODS**

**Cell lines:** Cell lines were authenticated by STR analysis (MD Anderson Cancer Center and at Fred Hutchinson Cancer Research Center) using STR markers, tested for interspecies contamination. U87MG STR profile was matched to the profile available at vendor site. GBM4 STR profile was determined using cells before and after genetic manipulations. Cells were tested routinely for mycoplasma contamination using Micoplasma alert kit (Lonza).

**RNA-seq:** Cell pellets were lysed in TRYZOL directly. Tumor fragments were grinded in liquid nitrogen using mortar and pestle followed by lysis in TRYZOL. Total RNA was isolated from TRIZOL lysates and standard RNAeasy (Qiagen) kit protocol. Single indexed TruSeq mRNA libraries were generated as outlined by the manufacturer using the TruSeq RNA library prep kit v2. The starting material for mRNA library construction consisted of 250 ng of total RNA and 5 µL of a 1:1000 dilution of ERCC spike-in Mix 2 in a total of 50 µL. Fragment size distribution and DNA concentration were measured using an Agilent Bioanalyzer and Qubit fluorometer, respectively. U87 TruSeq libraries were sequenced on the Illumina NextSeq 500 system using 40 bp paired end reads generating 25-30 million reads per library.

**Western Blotting**: Equal amounts of protein were separated on the SDS precast minigel (Bio-Rad), transferred to the PVDF membrane and hybridized with primary antibody TW (clone 2C1a; Santa Cruz), β-Actin (Sigma), phosphorylated and total AKT, (Cell Signaling, Santa Cruz), Periostin (Adipogen), Flag-tag (Rockland). Secondary HRP conjugated anti-rabbit and anti-mouse antibody and ECL reagent (Pierce) were used for antigen visualization. Cells were washed with ice-cold PBS and lysed in RIPA buffer on ice. Cell lysates were centrifuged, and equal amounts of protein were subjected to Western blotting.  In some cases, capillary electrophoresis was used to obtain protein expression levels. For capillary electrophoresis (WES) instrument was used according tor manufacturer instructions (ProteinSimple, San Jose, California, 95134 USA).

**Immunofluorescence:** Using freezing microtome (Leica) sections were prepared from paraformaldehyde fixed brain tumors exposed to 30% sucrose and embedded in OCT. Sections were blocked in Pro-block solution (Dianova) and hybridized with primary antibody (Flag-tag (Rockland), HuNu (Abcam) or Active Caspase 3-PE (BD-Biosciences) diluted in 50% Pro-block in PBS supplemented with 0.05% Tween 20, 0.05% Triton X100 (Sigma) overnight. For double staining antibody were applied sequentially. Appropriate secondary antibody Alexa Fluor 488 or 546 (Invitrogen) were used for antigen visualization.

**Quantitative real-time (qRT) PCR:** RNAeasy kit (Qiagen) with on column DNAse digest were used for total RNA extraction. Reverse transcription kit (Clontech) was used to reverse transcribe 1ug of total RNA. Quantitative PCR amplifications were performed using SYBR Green master mix in ABI PRISM 7900 sequence detection system (Applied Biosystems) and preset standard amplification cycle for 40 rounds. Following primers were used for POSTN amplification: forward-AATCATCCATGGGAACCAGA, reverse-ATTGGTGGGAGCAAAGAGTG and for TW amplification: forward-CGGGAGTCCGCAGTCTTA, reverse-CTTGAGGGTCTGAATCTTGCT. Amplification of target genes was normalized by GAPDH: forward-ATGTTCGTCATGGGTGTGAA, reverse-GTCTTCTGGGTGGCAGTGAT. The threshold exponential amplification cycle (Ct) was calculated by SDS system software. The specificity of amplifications was confirmed by amplicon melting profile.

**Supplemental figures:**

**Fig.S1**. **Growth advantage of TW expressing cells *in vivo:*** A) Western blot of TW protein in U87MG cells after CRISPR deletion of TW in U87MG cells with non-targeting (dNT) and two independent gRNAs targeting TW (dTW-A, dTW-B). Flag tagged Cas9 expression is detected with Flag antibody. B) Kaplan-Meier analysis demonstrating increased median survival of host mice implanted with TW deleted U87MG dTW-A cells (p=0.0004). C) Represent macroscopic images of GFP labeled terminal tumors. D) Western blot analysis of TW and Cas9 in cells before injection (lanes 1,2) and from independent isolates of dTW-A tumor derived cells (lanes 3-6) showing expression of TW and loss of Cas9 in dTW-A generated tumor cells. E) Flag immunostaining of Cas9 in control and U87MG dTW-A derived tumors confirms growth of undeleted tumor cells. T-tumor; NB-normal brain.

**Fig.S2** A) A-insertion generated by dTW-A targeting gRNA in TW confirmed by single cell sub-cloning and sequencing. B) Confirmation of TW protein loss in selected U87 sub-clones by Western blot.

**Fig.S3** Analysis of dTW-A target gRNA sequence for off-target effect. High risk off-target effects identified for USP35 and EXOC4 using proprietary algorithm (Kbiobox) based on proximity of mismatches (red) to PAM site (yellow). Arrows show high risk off target sequences with clustered mismatches.

**Fig.S4.** Representative histological appearance of terminal tumors derived from U87MG dNTsc and dTW-Asc (H&E staining). (scale bar =50μm).

**Fig.S5** **Inducible knockdown of TW in established tumors inhibits subsequent tumor growth**. A) Western blot analysis showing reduction of TW protein after doxycycline (DOX) induction of TW shRNA (U87 shTwi). B) Confirmation of reduced TW mRNA expression *in vivo*. TW expression was quantified by qRT-PCR in tumors generated from control and inducible shTW cells harvested after 3 and 5 days of DOX administration and the same cells after DOX exposure for 48 hours in vitro. Corresponding shScri samples accepted as 1. C) Macroscopic images of flank tumors harvested after 13 days of growth and 10 days of DOX supplemented diet. D) Volumetric comparison of control and shTWi tumor sizes showed significant effect of TW knockdown on growth of established tumors (p=0.0114).

**Fig.S6** Differentially expressed genes regulated by TW in U87MG cells *in vivo* and *in vitro*. Genes are listed in Table S3.

**Fig.S7**. Enlarged representation of Fig 2 C, D.

**Fig.S8** TCGA Level 3 data (given in array counts) was analyzed with respect to TW expression. A) TW expression distribution across 525 GBM samples from TCGA. B) Differences between low expressing (bottom 10%) and high expressing (top 10%) cohorts used for survival and DEG analysis. C) Difference in survival of high (dotted line) and low expressing (solid line) cohorts defined as above using TCGA RNA seq data.

**Supplemental Tables:**

**Table S1** Summary of single cell clones derived from U87MG cells and verified for mutations in TW and off-target high risk genes: EXOC4 and USP35

**Table S2** Summary of single cell clones derived from U87MG cells and verified for mutations in TWIST1

**Table S3** List of differentially expressed genes regulated by TW in U87MG cells *in vivo* and *in vitro*.

**Table S4** List of genes in Gene ontology categories regulated by tumor microenvironment and by TW status.

**Table S5** Summary of TW and POSTN expression correlation analyses in TCGA and RMBRANDT data sets. v
